# Accumulation of *α*-Synuclein in Cerebellar Purkinje Cells of Diabetic Rats and Its Potential Relationship with Inflammation and Oxidative Stress Markers

**DOI:** 10.1155/2017/5952149

**Published:** 2017-01-04

**Authors:** Volkan Solmaz, Hatice Köse Özlece, Hüseyin Avni Eroglu, Hüseyin Aktuğ, Oytun Erbaş, Dilek Taşkıran

**Affiliations:** ^1^Department of Neurology, Trakya University Faculty of Medicine, Edirne, Turkey; ^2^Department of Physiology, Kafkas University Faculty of Medicine, Kars, Turkey; ^3^Department of Histology and Embryology, Ege University Faculty of Medicine, Izmir, Turkey; ^4^Department of Physiology, Bilim University Faculty of Medicine, Istanbul, Turkey; ^5^Department of Physiology, Ege University Faculty of Medicine, Izmir, Turkey

## Abstract

*Objective.* The present study was conducted to evaluate the relationship between plasma oxidative stress markers such as malondialdehyde (MDA) and glutathione (GSH), inflammatory marker pentraxin-3 (PTX3), and cerebellar accumulation of *α*-synuclein in streptozotocin- (STZ-) induced diabetes model in rats.* Methods.* Twelve rats were included in the study. Diabetes (*n* = 6) was induced with a single intraperitoneal injection of streptozotocin (STZ, 60 mg/kg). Diabetes was verified after 48 h by measuring blood glucose levels. Six rats served as controls. Following 8 weeks, rats were sacrificed for biochemical and immunohistochemical evaluation.* Results.* Plasma MDA levels were significantly higher in diabetic rats when compared with the control rats (*p* < 0.01), while plasma GSH levels were lower in the diabetic group than in the control group (*p* < 0.01). Also, plasma pentraxin-3 levels were statistically higher in diabetic rats than in the control rats (*p* < 0.01). The analysis of cerebellar *α*-synuclein immunohistochemistry showed a significant increase in *α*-synuclein immunoexpression in the diabetic group compared to the control group (*p* < 0.01).* Conclusion.* Due to increased inflammation and oxidative stress in the chronic period of hyperglycemia linked to diabetes, there may be *α*-synuclein accumulation in the cerebellum and the plasma PTX3 levels may be assessed as an important biomarker of this situation.

## 1. Introduction

Diabetes mellitus (DM) is one of the most common metabolic diseases caused by insulin deficiency or resistance [1]. The disease may include all types of systemic involvement with morbidity affected most frequently by neurological complications [2]. In recent years, a growing body of evidence suggests a possible link between type 2 diabetes mellitus (T2DM) and neurodegenerative diseases such as Alzheimer's disease (AD) and Parkinson's disease (PD) [3–5]. In addition, it has been proposed that oxidative stress, advanced glycation end products (AGEs) formation, increased aldose reductase activity, and activated protein kinase C (PKC) have been involved in the molecular mechanisms underlying neurodegeneration in T2DM [3, 4, 6, 7].

Alpha-synuclein (*α*-synuclein), a protein localized to presynaptic terminals, binds synaptic vesicle membranes and contributes to vesicle trafficking and soluble NSF (N-ethylmaleimide sensitive factor) attachment protein receptor (SNARE) complex formation [8–10]. In recent years, several in vivo and in vitro studies have demonstrated that elevated *α*-synuclein levels could lead to its abnormal aggregation and neuronal degeneration [8–10]. Histopathologically, *α*-synuclein accumulates in the central nerve system (CNS) to form Lewy bodies in some diseases like idiopathic Parkinson's disease (PD), Lewy body dementia (LBD), and multisystem atrophy (MSA), called alpha-synucleinopathies [11, 12]. According to these studies, *α*-synuclein shows pathological accumulations in many regions of the CNS such as the cerebral cortex, cerebellum, and hippocampus. In addition, a very close relationship between *α*-synuclein, inflammation, and oxidative stress has been reported [13, 14].

Pentraxin-3 (PTX3) is an acute phase protein and a member of the pentraxin superfamily, which is recognized for its role in peripheral immunity and vascular inflammation in response to injury. Recently, increased levels of PTX3 in cerebrospinal fluid (CSF) and plasma samples have been found in neurodegenerative disorders such as PD and AD [15].

Although there are several studies that demonstrate biochemical and histopathological changes in the cerebrum including cortex and hippocampus in DM, there is still limited data regarding the relationship between cerebellar *α*-synuclein accumulation, oxidative stress, and inflammation in DM. Considering this, the present study was conducted to evaluate plasma oxidative stress markers such as malondialdehyde (MDA) and glutathione (GSH), inflammatory marker PTX3, and cerebellar accumulation of *α*-synuclein in streptozotocin- (STZ-) induced DM model in rats.

## 2. Materials and Methods

### 2.1. Animals

Twelve male Sprague-Dawley albino mature rats, 8 weeks of age, weighing 200–220 g, were used in the study. Animals were fed ad libitum and housed in pairs in steel cages in a temperature-controlled environment (22 ± 2°C) with 12-hour light/dark cycles. The experimental procedures were approved by the local ethics committee (KAU-HADYEK/2016-060). All animal studies strictly conformed to the animal experiment guidelines of the Committee for Human Care.

### 2.2. Chemicals

All chemicals were purchased from Sigma-Aldrich, Inc. (St. Louis, MO), unless otherwise noted.

### 2.3. Experimental Protocol

Diabetes was induced by intraperitoneal (i.p.) injection of STZ (Sigma-Aldrich, Inc.; Saint Louis, MO, USA) (60 mg/kg in 0.9% NaCl, adjusted to pH 4.0 with 0.2 M sodium citrate) for 6 rats (diabetes group, *n* = 6). Six rats served as control group and received no treatment. Diabetes was verified after 24 hours by evaluating blood glucose levels with the use of glucose oxidase reagent strips (Boehringer Mannheim, Indianapolis). The rats with blood glucose levels 250 mg/dL and higher were included in this study. Eight weeks later, the animals were euthanized and blood samples were collected by cardiac puncture to determine plasma glucose, MDA, GSH, and pentraxin-3 levels. Cerebellums were removed for *α*-synuclein immunohistochemistry.

### 2.4. *α*-Synuclein Immunohistochemistry

40 *μ*m thick cross sections were taken with a microtome (Leica MR 2145) from paraformaldehyde-fixed and paraffin-embedded cerebellum tissue. The sections were incubated with H_2_O_2_ (10%) for 30 min to eliminate endogenous peroxidase activity and blocked with 10% normal goat serum (Invitrogen) for 1 hour at room temperature. Subsequently, sections were incubated in primary antibodies (*α*-synuclein, Bioss Inc.; 1/100) for 24 h at 4°C. Antibody detection was performed with the Histostain-Plus Bulk kit (Invitrogen) against rabbit IgG, and 3,3′-diaminobenzidine (DAB) was used to visualize the final product. All sections were washed in PBS and photographed with an Olympus C-5050 digital camera mounted on an Olympus BX51 microscope. Brown cytoplasmic stained cells were scored as positive for *α*-synuclein immunostaining. The number of *α*-synuclein (+) cells was assessed by systematically scoring at least 100 Purkinje cells per field in 10 fields of tissue sections at a magnification of 100x.

### 2.5. Measurement of Lipid Peroxidation (MDA)

Lipid peroxidation was determined in plasma samples by measuring malondialdehyde (MDA) levels as thiobarbituric acid reactive substances [16]. Briefly, trichloroacetic acid and TBARS (thiobarbituric acid reactive substances) reagent were added to the plasma samples and then mixed and incubated at 100°C for 60 min. After cooling on ice, the samples were centrifuged at 3000 rpm for 20 min and the absorbance of the supernatant was read at 535 nm. MDA levels were calculated from the standard calibration curve using tetraethoxypropane and expressed as *μ*M.

### 2.6. Measurement of Plasma Glutathione (GSH) Levels

GSH content in plasma samples was measured spectrophotometrically according to Ellman's method [17]. In this method, thiols interact with 5,5′-dithiobis(2-nitrobenzoic acid) (DTNB) and form a colored anion with maximum peak at 412 nm. GSH levels were calculated from the standard calibration curve and expressed as *μ*M.

### 2.7. Evaluation of Plasma PTX3 Levels

Plasma pentraxin-3 (PTX3) levels were measured in each 100 *μ*L sample by standard ELISA apparatus at 450 nm using a PTX3 kit (Uscn Life Science Inc., Wuhan, China). PTX3 levels were determined in duplicate according to the manufacturer's guide. The detection range for PTX3 assay was 0.078–5 ng/mL.

### 2.8. Statistical Analysis

Data analyses were performed using statistical software (SPSS for Windows, Version 16, Statistical Package for the Social Sciences, Worldwide Headquarters SPSS Inc., Chicago, IL, USA). Statistical comparisons were completed using the Mann–Whitney *U* test. Results are given as mean ± SEM. A value of *p* < 0.05 was considered statistically significant.

## 3. Results

**Figure 1:**
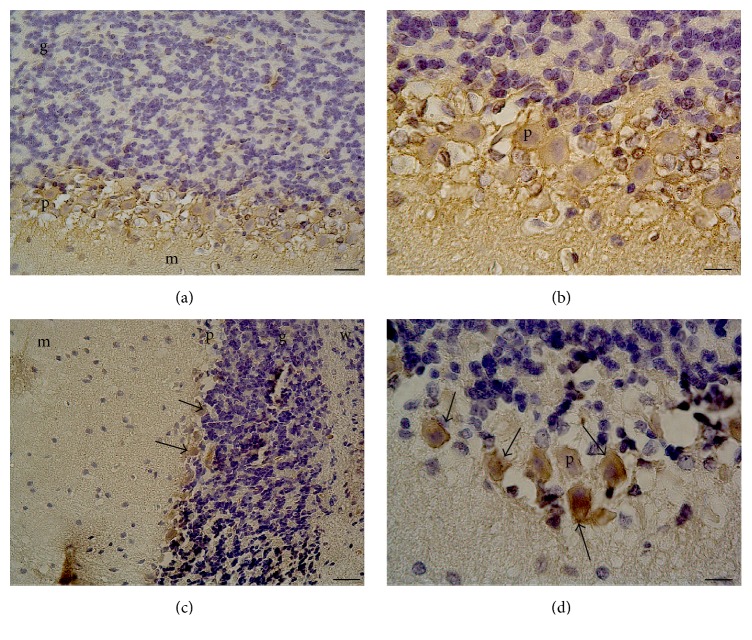
*α*-Synuclein immunoexpression in Purkinje cells of normal and diabetic rats. (a and b) Control rats cerebellum Purkinje cells (×20 and 100 magnification, resp.). (c and d) Diabetic rats cerebellum Purkinje cells (×10 and 100 magnification, resp.). p: Purkinje cell; g: granule cell layer; m: molecular layer; w: white matter. The arrows indicate *α*-synuclein (+) cells.

Table 1 shows the alterations in plasma glucose, MDA, GSH, and PTX3 levels in diabetic and control rats. The plasma glucose levels were significantly higher in the STZ-induced diabetic group than in the control group (*p* < 0.0001). The analysis of plasma oxidative stress parameters revealed significant differences between the groups. Plasma MDA levels were significantly higher in diabetic rats when compared with the control rats (*p* < 0.01), while plasma GSH levels were lower in the diabetic group than in the control group (*p* < 0.01). Also, plasma pentraxin-3 levels were statistically higher in diabetic rats than in the control rats (*p* < 0.01). The analysis of cerebellar *α*-synuclein immunohistochemistry showed a significant increase in *α*-synuclein immunoexpression in the diabetic group compared to the control group (*p* < 0.01) (Figure 1).

## 4. Discussion

The most important result obtained in our study is that oxidative stress marker (MDA) and inflammatory marker (PTX3) were significantly high in rats with hyperglycemia induced with STZ, and related to this, interestingly, *α*-synuclein immunoexpression was greater in the cerebellum of hyperglycemic rats. The present study is the first to show *α*-synuclein accumulation in Purkinje cells in the cerebellum of the diabetic rat model.

It is generally accepted that *α*-synuclein is found in the presynaptic membrane of neuronal tissues. However, recent studies have suggested that it is found intensely in mitochondria. Although the physiological function of *α*-synuclein is not fully understood, it is reported to have probable functions in synaptic plasticity, neurotransmitter release, neuronal differentiation, and regulation of neuronal viability [18, 19]. A variety of studies have shown that *α*-synuclein accumulates in Purkinje cells during various neurodegenerative diseases like PD and LBD [20, 21].

In recent years, hyperglycemia and neurodegeneration in the cerebellum have been studied in experimental models [3, 22–24]. In one of these studies, in a diabetic rat model induced with STZ, Baydas et al. found the astrocytic marker of glial fibrillary acidic protein (GFAP) and S100B levels were higher in hyperglycemic rats and related to this they reported neurodegenerative changes in many CNS regions such as cerebellum, hippocampus, and cerebral cortex [22]. Besides, a significant relationship between oxidative stress, inflammation, and neurodegeneration in DM has been frequently emphasized in recent studies; in fact, this situation has been named type 3 diabetes [25]. Studies related to this have reported increased incidence of Parkinson's disease in diabetic patients [6] and proposed that glycosylation of the *α*-synuclein protein due to hyperglycemia causes accumulation of Lewy bodies [26]. Previous experimental studies have mentioned a close relationship between increased oxidative stress and inflammation and accumulation of *α*-synuclein. For instance, a study by Wang et al. used ob/ob and db/db mice to create a diabetic model and observed that a single dose of 1-methyl-4-phenyl-1,2,3,6-tetrahydropyridine (MPTP) disrupted the insulin signaling not only in the pancreas and liver but also in the midbrain. Additionally, they revealed monomeric *α*-synuclein accumulation in both the pancreas and the substantia nigra in the midbrain. They proposed that this situation was related to systemic and central inflammation and neurotoxicity linked to hyperglycemia [27]. However, a study by Xie et al. reported that 1-acetyl-6,7-dihydroxyl-1,2,3,4-tetrahydroisoquinoline (ADTIQ) levels were elevated in both STZ-induced diabetic rats and transgenic *α*-synuclein gene PD mice model and this elevation was directly related to the accumulation of *α*-synuclein. Accordingly, they speculated that the relationship between hyperglycemia and PD might be related to ADTIQ [28]. In accordance with the previous studies, in our study, we observed significantly higher *α*-synuclein immunoexpression in the cerebellum of STZ-induced hyperglycemic rats compared to the control group.

Increased generation of reactive oxygen species (ROS) by hyperglycemia is recognized as a main cause of the clinical complications related to DM. In diabetes, not only overproduction of ROS but also reduced antioxidant defence mechanisms such as impaired GSH metabolism and alterations in antioxidant enzyme activities contribute to pathophysiology of hyperglycemia-induced cellular damage [29, 30]. MDA has been documented as a key biomarker of lipid peroxidation and oxidative stress [29–33]. Increased levels of plasma MDA in diabetics suggest an association between the high glycemic levels and oxidative damage in DM. The elevation in lipid peroxidation is also an indication of failure in cellular antioxidant defense mechanisms such as GSH [29–33]. GSH, a tripeptide, is considered as a biomarker of redox imbalance in all mammalian tissues. It defends cells against oxidative damage by maintaining SH groups of proteins in a reduced state and detoxifying reactive oxygen species (ROS) and also acting as a coenzyme in various enzymatic reactions. There are several clinical and experimental studies revealing the elevated MDA and decreased GSH levels in DM [31–33]. In our study, plasma MDA levels were found to be significantly high in diabetic group whereas GSH levels were diminished compared to control group. In agreement with previous studies, the findings of the present study suggest that exposure to prolonged durations of hyperglycemia may enhance lipid peroxidation and suppress GSH levels as a result of increased consumption of NADPH due to activation of polyol pathway by glucose.

The acute phase glycoprotein of PTX3 has been previously investigated for its relationship with many chronic diseases including DM [34, 35]. Additionally, it has been reported that the levels of acute phase protein increased in a variety of neurodegenerative diseases [36, 37]. For instance, Lee et al. have observed high plasma PTX3 levels in idiopathic PD patients and found significant correlation between the PTX3 levels and daily life activities and motor functions [38]. According to the findings of the previous studies combined with the results of the current experiments, we can speculate that PTX3, apart from being an acute phase protein, may be considered as a possible biomarker of increased inflammation in the chronic period of systemic and neurodegenerative diseases.

Taken together, to the best of our knowledge, our study is the first to demonstrate *α*-synuclein accumulation in the cerebellum and increased plasma PTX3 levels in the STZ-induced diabetes model. In conclusion, due to increased inflammation and neurotoxicity in the chronic period of hyperglycemia linked to diabetes, there may be *α*-synuclein accumulation in the cerebellum and the plasma MDA, GSH, and PTX3 levels may be assessed as important biomarkers of this situation. However, future experimental and clinical studies are required to clarify the molecular mechanisms underlying cerebellar neurodegeneration in DM.

## Figures and Tables

**Table 1:** Plasma glucose, glutathione (GSH), malondialdehyde (MDA), and pentraxin-3 (PTX3) levels in control and diabetics rats at the end of the study.

	Glucose (mg/dL)	GSH (*μ*M)	MDA (*μ*M)	PTX3 (ng/mL)	*α*-Synuclein immunoexpression (%)
Control (*n* = 6)	98.6 ± 8.4	13.58 ± 1.25	0.18 ± 0.06	1.24 ± 0.19	3.48 ± 0.4
Diabetic rats (*n* = 6)	485.9 ± 35.23^*∗∗*^	1.76 ± 0.32^*∗*^	0.39 ± 0.02^*∗*^	2.65 ± 0.04^*∗*^	85.62 ± 5.84^*∗∗*^

Data are expressed as mean ± SEM.

^*∗*^ *p* < 0.01, ^*∗∗*^*p* < 0.0001, control group compared with diabetic rats.
